# Eco-Friendly Extraction and Characterisation of Nutraceuticals from Olive Leaves

**DOI:** 10.3390/molecules24193481

**Published:** 2019-09-25

**Authors:** Cinzia Benincasa, Ilaria Santoro, Monica Nardi, Alfredo Cassano, Giovanni Sindona

**Affiliations:** 1 CREA Research Centre for Olive, Citrus and TreeFruit, C.da Li Rocchi, I-87036 Rende, Cosenza, Italy; 2 Dipartimento di Chimica, Università della Calabria, Cubo 12C, I-87036 Rende, Cosenza, Italy; ilaria.santoro@unical.it (I.S.); giovanni.sindona@unical.it (G.S.); 3 Dipartimento di Scienze della Salute, Università Magna Græcia, Viale Europa, I-88100 Germaneto, Cosenza, Italy; monica.nardi@unicz.it; 4 Institute on Membrane Technology, ITM-CNR, c/o University of Calabria, via P. Bucci, 17/C, I-87036 Rende, Cosenza, Italy; a.cassano@itm.cnr.it

**Keywords:** green chemistry, olive leaves, natural products, tandem mass spectrometry

## Abstract

Olive tree (*Olea europaea* L.) leaf, a waste by-product of the olive oil industry, is an inexpensive and abundant source of biophenols of great interest for various industrial applications in the food supplement, cosmetic, and pharmaceutical industries. In this work, the aqueous extraction of high-added value compounds from olive leaves by using microfiltered (MF), ultrapure (U), and osmosis-treated (O) water was investigated. The extraction of target compounds, including oleuropein (Olp), hydroxytyrosol (HyTyr), tyrosol (Tyr), verbascoside (Ver), lutein (Lut), and rutin (Rut), was significantly affected by the characteristics of the water used. Indeed, according to the results of liquid chromatography tandem mass spectrometry, the extracting power of microfiltered water towards rutin resulted very poor, while a moderate extraction was observed for oleuropein, verbascoside, and lutein. On the other hand, high concentrations of hydroxytyrosol were detected in the aqueous extracts produced with microfiltered water. The extraction power of ultrapure and osmosis-treated water proved to be very similar for the bio-active compounds oleuropein, verbascoside, lutein, and rutin. The results clearly provide evidence of the possibility of devising new eco-friendly strategies based on the use of green solvents which can be applied to recover bioactive compounds from olive leaves.

## 1. Introduction

In recent years, fundamental research has focused on using resources found in the environment for the protection of people’s well-being [1]. Plant materials are widely used to maintain human health. Traditional medications, food supplements, and functional foods typically contain antioxidant compounds which may inhibit or decrease the rate of oxidation of other molecules by preventing the initiation and/or propagation of the chain reaction of free radicals [2]. Considering that plant materials are extremely complex matrices comprised of many components that can interfere with good separation, classic extraction procedures often involve different steps and the use of unsustainable solvents. Furthermore, the starting matrix from which these compounds are extracted is not always a waste product in the biomass processing industry. The development of green and environmentally friendly extraction methods of natural products is a hot research topic in the area of chemistry and technology [3]. In several experimental studies, phenols have demonstrated a wide spectrum of pharmacological activities beyond their antioxidant properties [4,5,6]. A potential source of these compounds is found in olive leaves: they are grouped with regard to major molecular characteristics as simple phenols and acids, lignans, and flavonoids [7] including flavones (luteolin-7-glucoside, apigenin-7-glucoside, diosmetin-7-glucoside, luteolin, and diosmetin), flavonols (rutin), flavan-3-ols (catechin), substituted phenols (tyrosol, hydroxytyrosol, vanillin, vanillic acid, and caffeic acid) [8,9], oleuropein, and other secoiridoids [10,11]. The latter are exclusive to the Oleaceae family. In fact, secoiridoids and other derivatives are the principal compounds of olive leaves [12], among which a major compound that is frequently reported is oleuropein. Flavonoids may occur in appreciable amounts [13] while simple phenols and acids are present in lower amounts.

The recovery of bioactive molecules from plant extracts, in view of the accepted use of these natural compounds as nutraceuticals [14,15,16,17,18,19,20], has gained a grown interest in the last decades. Nutraceuticals are commercially available and in great demand [21,22] as they possess the special role of preventing or even supporting medical therapies [23,24,25]. A very large number of studies describe the recovery of phenols from plant tissues [9,10,11,26,27], but all known methods applied to the extraction of phenols from leaves are based on the use of solvents, supercritical fluids, and classical analytical techniques using maceration assisted by liquid solvents [28]. Other methods can reduce solvent consumption or can use green solvents representing an environmental and economical alternative [15,19,29]. In recent years, concerns about the environmental impact have emerged as an issue of priority in society. New aspects related to the use of agro-industrial residues as by-products for further exploitation of high-value products are increasingly gaining interest, and their recovery may be economically attractive. Advances in biotechnology potentially offer opportunities for economic utilization of plant food residues such as grape and olive pomace, leaves, barks, roots, etc. The idea of turning ‘‘waste to wealth” by means of industrial food residues can considerably contribute to sustainable development. The by-products of the olive oil industry are an extraordinary source of bioactive phenolic compounds [19]. In this context, the concept of “Green Chemistry” has great importance in industrial processes to reduce or eliminate the use and generation of hazardous substances and was developed in principle to guide the chemists in their search towards greenness [30]. In the last decade, a new generation of green solvents and green methodologies to be used both in synthetic transformations and in extraction processes has been developed [31,32,33].

The use of water as solvent has attracted much interest in recent years [34,35]. Water features many benefits: water itself is not expensive, it can potentially improve reactivity and selectivity and enable the recycling of the catalyst [36,37,38], and it can allow mild reaction conditions in the use of protecting groups [39,40] and in the synthesis of bio-active compounds [41,42]. The study chemistry in water has also been an interesting way to gain insights into the biosynthesis and extraction of natural products [14,43,44].

To the best of our knowledge, the water-based extraction of phenolic compounds from olive leaves has been poorly investigated. Goldsmith et al. [45] used the response surface methodology (RSM) approach in order to identify the best possible combination of temperature, extraction time, and sample-to-solvent ratio for the aqueous extraction of phenolic compounds from olive leaves. The optimal conditions were proposed to be at 90 °C for 70 min at a sample-to-solvent ratio of 1:60 g/mL.

Ansari et al. [46] developed a green and inexpensive water-based procedure to extract oleuropein from olive leaf samples. The experimental results revealed that deionised water adjusted to pH 3 at 60 °C for 4 h had the highest extraction efficiency.

In this work we aimed to evaluate the aqueous extraction of bio-active compounds from whole and chopped olive leaves, such as oleuropein (Olp), hydroxytyrosol (HyTyr), tyrosol (Tyr), verbascoside (Ver), lutein (Lut), and rutin (Rut), by using ultrapure, microfiltered and osmosis-treated water. Tandem mass spectrometry analyses, extensively used in the field of structure evaluation of natural products, were performed to fully characterize the phenolic compounds of olive leaves.

## 2. Results and Discussion

### 2.1. Concentration and Trend of Bio-Active Compounds in Aqueous Extracts of Chopped Olive Leaves

**Figure 1:**
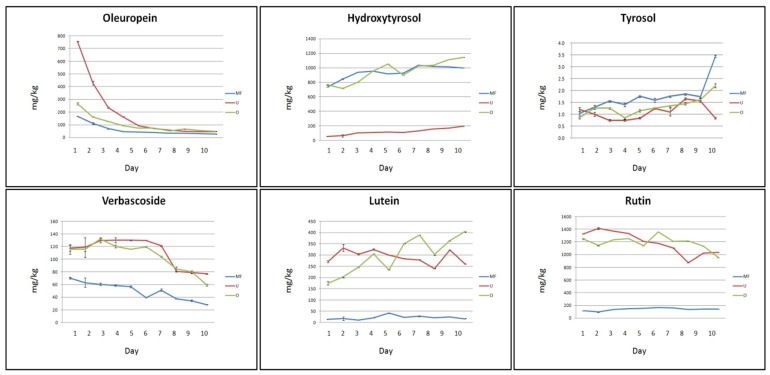
Trend of the concentrations of bio-active compounds in aqueous extracts of chopped olive leaves. Three types of water were tested: ultrapure (U), microfiltered (MF), and osmosis-treated (O) water. Data are expressed as the means ± standard error of the mean (SEM) of three independent observations. Statistical results from one-way analysis of variance (ANOVA) followed by Tukey-test are provided in Table S1 in Supporting Information.

Experimental results obtained from the aqueous extraction of chopped olive leaves are graphically summarized in Figure 1.

Water properties are very important for the extraction of Olp in chopped leaves: in particular, ultrapure (U) water appears to stimulate the migration process of Olp from the leaves to the solution more than micro-filtered (MF) and osmosis-treated (O) water. In fact, the extracting power of O and MF water was three and five times less than the extracting power of U water, respectively. In addition, for each type of water used for the extraction, the Olp concentration was affected by the time of infusion of the leaves: it reached its maximum concentration in the first day of infusion (753, 268, and 170 mg/kg in U, O, and MF water, respectively) and decreased up to reach a constant value after six days of infusion. The recovery of Olp resulted much higher if compared with some data reported in literature. Indeed, the use of water alone did not result in any detectable signal for Olp yield quantification as reported by Cifà et al. [47] where different pH of the water, times, and temperatures were tested. The authors, contrarily to us, finally established that water was not a good solvent to extract Olp from olive leaves. The Olp concentration in MF, O, and U water resulted also much higher than that reported by Ghomariet al. [48] when cold distilled water was used for the extraction (about 100 mg/kg). On the other hand, higher Olp concentrations were detected by using distilled water at 60 °C (19.3 ± 0.99 mg/g) and distilled water at 60 °C and pH 3 (23.36 ± 0.91 mg/g) [48]. Similarly, Ansari et al. [40] reported that distilled water at 60 °C and pH 3 for 4 h could allow the extraction of a large amount of Olp. In addition, Malik and Bradford [49] reported that although extraction in 80% methanol is the most effective method for olive leaf polyphenols, boiling of dried leaves was also a very efficient method for extracting Olp and Ver that gave 96% and 94% recoveries of these compounds, respectively, when compared with the methanol extract.

A prevalence of HyTyr in comparison with other bioactive compounds was detected in all aqueous extracts. These results are consistent with those reported by Herrero et al. [44] who showed that HyTyr was the main phenolic component on the water pressurized liquid extraction olive leaves extracts when water is used as extracting agent. On the other hand, oleuropein was the main component in the extracts obtained with ethanol. However, the extracting power of O and MF water resulted quite similar and almost ten times greater than the extracting power of U water (768, 736 and 56 mg/kg, respectively on the first day of infusion). HyTyr concentration slowly increased throughout the experimental period to reach high values at the end of the process (1139, 1008, and 189 mg/kg in O, MF, and U water, respectively). The recovery of HyTyr by using cold distilled water according to Ghomari et al. [48] was 200 mg/kg corresponding to our value at the end of the process with U water. The release of Tyr was, instead, very poor during all the experimental period and none of the three waters produced a significant difference (3.6, 2.2, and 1 mg/kg in MF, U, and O water, respectively, on the tenth day of infusion). These results were in agreement with those reported in the literature [48].

U and O water showed a similar extracting power towards Ver, almost twice when compared to the extracting power of MF water (117 mg/kg in U and O water, 68 mg/kg in MF water, on the first day of infusion). The concentration of Ver reached its highest value on the sixth day of infusion in U water and on the third day of infusion in O water (133 and 134 mg/kg, respectively). Ver concentration decreased over the time to almost half its value by the end of the process (77, 57, and 27 mg/kg in U, O, and MF water, respectively). Ver resulted undetectable in the extraction by maceration in cold distilled water, as reported by Ghomari et al. [48]. A similar behaviour was observed for Lut: the migration process of this flavone from the leaves to the water was bland in MF water (15 and 18 mg/kg were the value registered from the start to the end of the infusion process) and more vigorous in U and O water (277and 178 mg/kg, respectively). The value of its concentration was higher on the sixth day of infusion in U water and on the last day of infusion in O water (356 and 400 mg/kg, respectively). The recovery of this flavone in olive leaves extracts obtained by maceration with distilled cold water was ten times lower (about 30 mg/kg) [48].

The release of Rut resulted very low in MF water during all the experimental process (between 91 and 175 mg/kg). On the other hand, the extracting power of U and O water was very incisive from the first day of infusion (1331 and 1244 mg/kg, respectively). Moreover, the concentration of Rut was not affected by the infusion time. The amount of rutin extracted in this study was much higher than that recorded by Ghomari et al. [48] who reported concentrations between 200 and 500 mg/kg.

The overall results indicated that with the exception of HyTyr and Tyr, the use of O and U water produced higher extraction efficiency of bioactive compounds, probably due to the absence or reduction of salt compounds which can affect the extraction process.

### 2.2. Concentration and Trend of Bio-Active Compounds in Aqueous Extracts of Whole Olive Leaves

**Figure 2:**
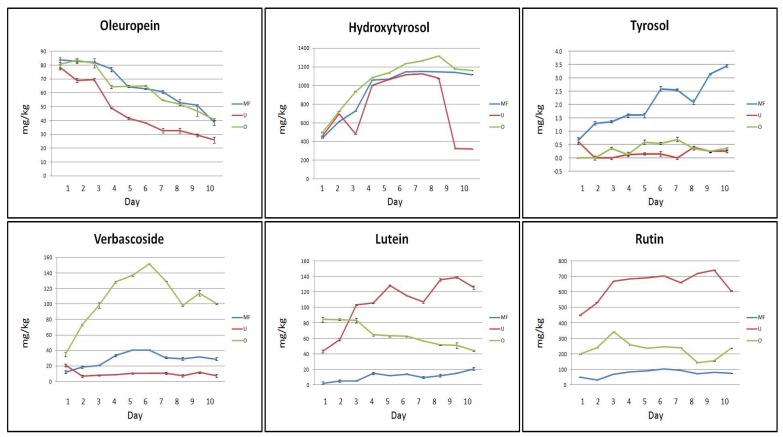
Trend of the concentrations of bio-active compounds in aqueous extracts of whole olive leaves. Three types of water were tested: ultrapure(U), microfiltered (MF), and osmosis-treated (O) water. Data are expressed as the means ± SEM of three independent observations. Statistical results from one-way analysis of variance (ANOVA) followed by Tukey-test are provided in Table S1 in Supporting Information.

As expected, the results obtained from the analysis of whole leaf infusions provided a concentration of bio-active compounds much lower than in the infusions discussed above. Figure 2 shows the trend of the concentrations of the bio-active compounds recorded in the whole olive leaves during the extraction process.

The migration of some bio-active compounds from whole leaves to the solution was not affected by the quality of the water. In particular, the extracting power of MF, U, and O water towards Olp was similar both at the beginning (86 mg/kg for MF and O water, and 81 mg/kg for U water) and at the end of the process (41 mg/kg for MF and O water, and 27 mg/kg for U water). Similarly, the extracting power of selected waters towards HyTyr resulted to be very similar at the beginning of the process (441, 467, and 512 mg/kg in MF, U, and O water, respectively, on the first day of infusion). HyTyr concentration reached the highest value on the sixth day of infusion in MF water (1152 mg/kg), on the seventh day of infusion in O water (1128 mg/kg), and on the eighth day of infusion in U water (1313 mg/kg).

As for chopped olive leaves, a prevalence of HyTyr in comparison with other bioactive compounds was detected in all aqueous extracts. These results are in agreement with those reported by Herrero et al. [50]. On the other hand, the release of Tyr was, again, very poor or null during all the experimental period, regardless of the type of water employed for the maceration process (3.4, 0.1, and 0.2 mg/kg in MF, U, and O water, respectively, on the tenth day of infusion).

The recovery of Ver, Lut, and Rut resulted to be influenced more greatly by the type of water. The concentration of Ver reached its highest value on the first day of infusion in U water (21 mg/kg) and on the sixth day of infusion in MF and O water (39 and 148 mg/kg, respectively). The migration process of Lut from the leaves to the water resulted very bland in MF water (3 and 19 mg/kg were the values registered from the start to the end of the infusion process) and gentle in U and O water (45 and 86 mg/kg, respectively). The concentration of the flavone constantly increased in U water, but decreased in O water (127 and 41 mg/kg, respectively). Similarly, the release of Rut resulted very low in MF water during the whole extraction process (between 34 and 112 mg/kg). The extracting power of U and O water, in contrast, gave some results from the first day of infusion (453 and 203 mg/kg, respectively). However, the concentration of the flavonol remained constant, or slightly increased, by the end of the process (610 and 239 mg/kg in U and O water, respectively).

The experimental results clearly indicated that the infusions of chopped leaves were richer in bio-active compounds in comparison to those of whole leaves. This could be explained by considering that the integrity of cell membranes of whole leaves does not promote the release of these bio-active compounds in water [51]. Furthermore, the glycosidic molecules such as Olp and Ver, even if extracted in important concentrations from the first day of infusion, were less stable over time compared to the other bio-active compounds analysed. This behaviour is probably due to the action of deglycosylation enzymes belonging to the leaves and released during the maceration process [52]. On the other hand, the migration process of HyTyr and Lut from the leaves to the solution has always shown to be increasing over time, while the extraction of Rut took place immediately and remained constant throughout the experimentation period.

The characteristics of the water used for the extraction processes were very important for the success of the extraction itself. In fact, for the flavonol Rut the extraction power of the MF water was very poor, while it was bland for Olp, Ver, and Lut. A completely different situation was found for HyTyr which, in MF water, was recovered in high concentrations. 

The extraction power of U and O water proved to be very similar for the bio-active compounds Olp, Ver, Lut, and Rut. These waters have very similar chemical characteristics: U water contains by definition only H_2_O, and H^+^ and OH^−^ ions in equilibrium; osmosis-treated water is minimally mineralized. MF, instead, purified of chlorine and derivatives, dust and rust, preserves the mineral salts. These could in some way interfere with the process of migration of bio-active compounds (Rut), disturbing their accumulation in solution (Olp, Ver, and Lut), or even encouraging it (HyTyr).

### 2.3. Method Validation

**Figure 3:**
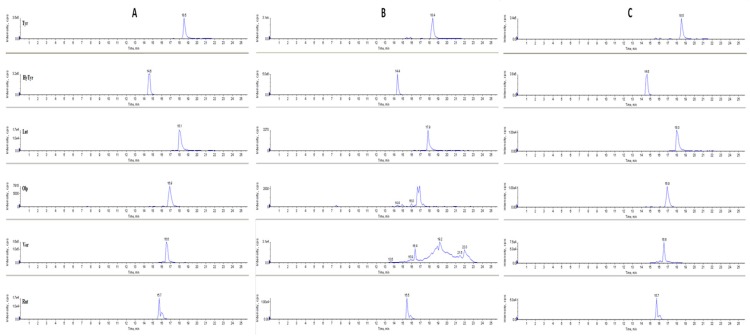
HPLC-MS chromatograms in multiple reactions monitoring MRM mode of bio-active compounds in the aqueous solutions of whole olive leaves after five days of maceration with: ultrapure water (**A**); microfiltered water (**B**) and osmosis-treated water (**C**).

For the determination of the best instrumental conditions, standard solutions of the selected bio-active compounds were introduced directly into the ion source of the mass spectrometer by direct infusion (FIA) at a flow rate of 10 μL/min. The mass spectrum obtained for Olp, a glycosylated secoiridoid, showed a pseudo molecular ion at *m/z* 539 and ionic fragments at *m/z* 307 and 275. These two characteristic ionic fragments originate from the ion at *m/z* 377 (a molecule resulting from the breakdown of the glycosidic bond of oleuropein which in ESI-MS experiments produces the ion at *m/z* 307 from the loss of a C_4_H_6_O fragment), and the ion at *m/z* 275 (derived from a rearrangement of other fragments). The mass spectrum obtained for HyTyr showed the deprotonated molecule [M − H]^−^ at *m/z* 153 and the ionic fragment at *m/z* 123 due to the loss of a CH_2_OH molecule. The mass spectrum obtained for Tyr did not produce any important fragments; therefore, the MRM measurements were conducted by scanning in both quadrupoles the deprotonated molecule [M − H]^−^ at *m/z* 137. The mass spectrum obtained for Ver, a phenylpropanoid and an ester sugar of caffeic acid, showed an intense peak corresponding to the deprotonated molecule [M − H]^−^ at *m/z* 623 and two characteristic ionic fragments at *m/z* 461 and 161. The loss of caffeic acid in fact produces an ion at *m/z* 461 and a neutral fragment, while the peak at *m/z* 161 results from a proton transfer and from the production of an anionic ketene. The mass spectrum obtained for Lut showed the deprotonated molecule [M − H]^−^ at *m/z* 285 and fragments at *m/z* 133 and 151corresponding to retro Diels Alder fragmentation of flavone molecule. The mass spectrum obtained for the flavonol Rut showed the deprotonated molecular ion at *m/z* 609 and a fragment at *m/z* 301 which is diagnostic of quercetin derivatives. The MRM transitions monitored for the assay of the bio-active compounds under investigation were, therefore, as follows: 539 → 307 and 539 → 275 for Olp; 153 → 123 for HyTyr; 137 → 137 for Tyr; 623 → 161 and 623 → 461 for Ver; 285 → 133 for Lut; 609 → 301 for Rut (Figure S1 in Supporting Information). Calibration curves constructed using a least-squares linear regression analysis were linear with correlation coefficients of 0.998 and 0.999 (Figure S2 in Supporting Information). Subsequently, to determine the best chromatographic conditions, the same standard solutions were injected into the HPLC-MS system through the chromatographic column (Figure 3). Spike solutions at 25 and 50 µg/mL gave good recoveries in a range between 87% and 109%, and satisfactory precision with relative standard deviation (RDS) in a range between 0.038% and 0.207% (Table 1). All data were obtained from three independent injections.

## 3. Materials and Methods 

### 3.1. Sample Collection and Preparation

Olive leaves from Coratina cultivar were collected from plants belonging to the olive grove of CREA-Research Centre for Olive, Citrus and TreeFruit, located in Rende (CS) and placed at 204 meters a.s.l. (39°22′17.681′′N, 16°13′58.342′′E).

Immediately after the harvest, plant tissues were processed for further experiments. Both whole and leaves roughly chopped by hands were considered in order to evaluate the best conditions for the extractions. In particular, 6 g of sample were placed in stoppered flasks containing 50 mL of water and kept at room temperature. The maceration processes were monitored by collecting each day, for 10 days, aliquots of the solutions and by performing high performance liquid chromatography tandem mass spectrometric analyses (LC-MS/MS). The maceration process of olive leaves was carried out by applying low-cost and eco-friendly methods where no solvents and supercritical fluids were used. 

Three types of water were tested: ultrapure (U), microfiltered (MF), and osmosis-treated (O) water. U water was produced through a Milli-Q plus water purification system (Millipore, Bedford, MA, USA) which includes a four-cartridge purification pack (Qpak), using deionized water as feed water. This module includes activated-carbon and ion-exchange resins housed within four polypropylene cylinders. A final filter (pore size 0.22 μm), within polycarbonate housing, was part of the dispensing point of this unit. The produced water was characterized by an electrical conductivity of 0.055 μS/cm. MF and O water were obtained from tap water. MF was performed by using a bench laboratory plant consisting of a stainless steel feed tank, a magnetic drive gear pump and a stainless-steel cell able to accommodate a flat-sheet membrane with a surface area of 38.46 cm^2^. The cell was equipped with a polyvinylidene fluoride membrane (MV020 T) with a nominal pore size of 0.2 μm, supplied by Microdyn-Nadir GmbH (Wiesbaden, Germany). Transmembrane pressure (TMP) was measured by two manometers allocated before and after the membrane cell and regulated by a pressure control valve on the concentrate outlet. Crossflow velocity (CFV) was controlled by a digital flowmeter. Temperature was controlled by using a cooling system fed with tap water and monitored by a digital thermometer inserted in the feed tank. Tap water was microfiltered at a TMP of 0.5 bar and an operating temperature of 25 ± 2 °C, producing a permeate water stream with an electrical conductivity of about 810 μS/cm. Reverse osmosis (RO) was performed by using a RO laboratory bench plant consisting of a control panel, a cylindrical jacketed feed tank (with a capacity of 5 L) constructed from stainless steel (SS 316), a feed plunger pump with belt drive (Cat Pumps, Milano, Italy, Model 3CP1221), two pressure gauges (Wika Instrument, Lawrenceville, GA, USA) (max pressure 100 bar, absolute error 1 bar), a digital flow meter (SM6000, ifm electronic gmbh, Essen, Germany), a thermometer placed inside the feed tank, and a cylindrical housing able to accommodate a 11.74 × 1.75-inchspiral-wound membrane module. The adjustment of operating pressure and feed flow rate was done by simultaneously pump rotation control through a frequency inverter and a needle valve. The operating temperature was controlled by circulating a coolant (cold water) through the tank jacket. The plant was equipped with a thin-film polyamide membrane module (SC1812-34D), supplied by GE Water & Process Technologies (Hopkins, MN, USA), having a NaCl rejection of 99.5% and a membrane surface area of 0.32 m^2^. The system was operated at a TMP of 6 bar and an operating temperature of 25 ± 2 °C producing a permeate water stream with an electrical conductivity of 42.7 μS/cm.

### 3.2. Quantitative Analysis 

Standards of oleuropein (Olp), tyrosol (Tyr), and 3-hydroxytyrosol (HyTyr) were purchased from Sigma–Aldrich (Sigma-Aldrich, St. Louis, MO, USA); verbascoside (Ver), lutein (Lut), and rutin (Rut) were purchased from Extrasynthese (Genay, France). All solvents were of LC/MS grade and purchased from VWR International. The assay of the bio-active analytes was achieved by external standard calibration: standard stock solutions were prepared by dissolving reference compounds in ethanol. Aliquots of these solutions were further diluted with water/0.1% formic acid to obtain calibration standards at concentrations between 1 and 200 µg/mL for Olp and HyTyr, 1 and 100µg/mL for Tyr, Lut, and Rut, and 1 and 150 µg/mL for Ver. The performance of the experiments was checked by recovery tests of spike solutions (Table 1).

### 3.3. High-Performance Liquid Chromatography/Tandem Mass Spectrometry

HPLC analysis were performed using an Agilent Technologies 1200 series liquid chromatography system equipped with G1379B degasser, G1312A pump, and G1329A autosampler. The chromatographic separation was achieved by injecting 10 µL of ethanol extract in an Eclipse XDB-C8-A column (5 µm particle size; 150 mm length; 4.6 mm internal diameter) (Agilent Technologies, Santa Clara, CA, USA) and a mobile phase consisted of an aqueous formic acid (0.1%) solution (A) and methanol (B). The separation of the analytes was achieved in 25 min at a flow rate of 350 µL/min. In particular, the elution profile was as follows: 0–10 min, 10–100% B (*v/v*); 10–12 min, 100% B; 12–20 min, 100–10% B (*v/v*); and 20–25 min, 100–10% B (*v/v*). The time for the column to re-equilibrated was 5 min. The mass spectrometer utilized for MS/MS analyses was an API 4000 Q-Trap (Applied Biosystem/MSD Sciex, Foster City, CA, USA). Each compound was monitored in negative ion mode using multiple reaction monitoring (MRM). The instrumental parameters, such as entrance potential (EP), declustering potential (DP), collision energy (CE), and collision exit potential (CXP) were, therefore, optimized for each transition monitored. Ionspray voltage (IS) was set at –4500 V; curtain gas at 20 psi; temperature at 400 °C; ion source gas(1) at 35 psi; ion source gas(2) at 45 psi; and collision gas thickness (CAD) medium. The HPLC-MS chromatograms of the bioactive compounds analysed in MRM mode are shown in Figure 3. In particular, the chromatograms refer to the analysis of the analytes of interest in the aqueous solutions of whole olive leaves after five days of maceration with: ultrapure water (A), microfiltered water (B) and osmosis-treated water (C). The retention times, expressed in minutes (min) are shown on the abscissas, whilst the intensity signals, expressed as counts per scan (cps), on the ordinates.

### 3.4. Statistical Analysis

The results are expressed by mean ± standard error of the mean (SEM) from at least three independent experiments. For statistical comparisons, quantitative data were analysed by one-way analysis of variance (ANOVA) followed by Tukey-test according to the statistical program SigmaStat1 (Jandel Scientific, Chicago, IL, USA). As the *p*-values were less than 0.001, there is a strong evidence for significant differences between the aqueous extraction of bio-active compounds from whole and chopped olive leaves by using ultrapure, microfiltered and osmosis-treated water (Table S1 in Supporting Information). 

## 4. Conclusions

**Figure 4:**
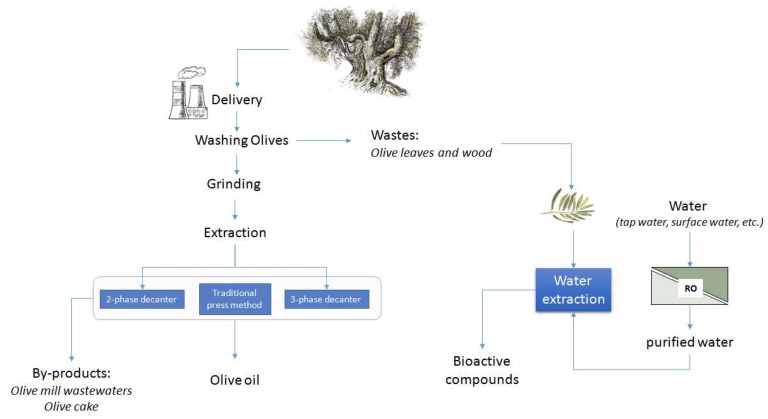
Scheme of olive oil production and aqueous extracts of highly nutritional and pharmacological value from olive leaves (RO, reverse osmosis).

The agro-food industries are focused on the use of bio-active compounds and nutraceuticals obtained from industrial waste products. Even though conventional extraction still is the main approach for obtaining bio-active compounds, this technology is not aligned with “green” and sustainable production, as it is very often accompanied by high expenditure and disposal of energy and toxic chemicals. The eco-friendly separation of bio-compounds from agro-industrial waste, supported by the use of an eco-sustainable and low-cost solvent such as water, is obviously attractive from both socio-environmental and economic points of view. Opening up new occasions for ecological approaches designed for bio-economy and circular economy models, the future food manufacturing can foresee better solutions for industrial production and applications. With the aim to increase production and process efficiency, reducing solvent and energy consumption and decreasing food waste by improving shelf life, may certainly have an important impact in changing industrial and academic practices. The previously discussed results clearly provide evidence for the possibility of devising new eco-friendly strategies that can be applied to recover important active compounds from olive leaves. In fact, from the sole use of water it was possible to obtain extracts of molecules of great health and pharmacological value by using a cheap and renewable source of natural product that is also an agricultural and industrial waste. In the procedure described, the use of waste material and the eco-sustainable and easily available solvents are proposed to contribute to sustainable development (Figure 4). The results that have been achieved were the followings: the infusions of chopped leaves were richer in bio-active compounds than infusions of whole leaves. This could be explained by considering that the integrity of cell membranes of whole leaves does not promote the release of these bio-active compounds in the water solutions. Furthermore, the glycosidic molecules such as Olp and Ver, even if extracted in important concentrations from the first day of infusion, were less stable over time compared to the other bio-active compounds analysed. This behaviour is probably due to the action of deglycosylation enzymes belonging to the leaves and released during the maceration process. On the other hand, the migration process of HyTyr and Lut from the leaves to the solution has always been shown to increase over time, while extraction of Rut took place immediately and remained constant throughout the experimentation period. The characteristics of the water used for the extraction processes were very important for the success of the extraction itself. In fact, for the flavonol Rut the extraction power of the MF water was null, while it was bland for Olp, Ver, and Lut. A completely different situation was found for HyTyr which, in MF water, was recovered in high concentrations. The extraction power of U and O water proved to be very similar for the bio-active compounds Olp, Ver, Lut, and Rut. These latter waters have very similar chemical characteristics: ultrapure water contains by definition only H_2_O and H^+^ and OH^-^ ions in equilibrium; osmosis-treated water is minimally mineralized. The MF water, purified of chlorine and derivatives, dust, and rust, preserves the mineral salts. These could in some way interfere with the process of migration of bio-active compounds (Rut), disturbing their accumulation in solution (Olp, Ver, and Lut), or even encouraging it (HyTyr). 

## Figures and Tables

**Table 1:** Results from recovery tests of spike solutions at 25 and 50 µg/mL. Data are expressed as the means ± relative standard deviation (RSD) of three independent observations. Olp: oleuropein; HyTyr: hydroxytyrosol; Tyr: tyrosol; Ver: verbascoside; Lut: lutein; Rut: rutin.

	Spiked Solution (25 µg/mL)		Spiked Solution (50 µg/mL)	
	Found	Recovery	Found	Recovery
Analyte	mean ± RSD	%	mean ± RSD	%
Olp	23.333 ± 0.108	93	50.667 ± 0.063	99
HyTyr	25.010 ± 0.080	100	52.333 ± 0.058	104
Tyr	24.333 ± 0.207	97	49.667 ± 0.081	99
Verb	24.667 ± 0.062	99	54.667 ± 0.038	109
Lut	21.667 ± 0.133	87	49.667 ± 0.111	99
Rut	22.512 ± 0.092	90	51.667 ± 0.095	103

## Supplementary Materials

The following are available online, Figure S1: LC-MS/MS spectra of the main phenolic compounds analysed: oleuropein (Olp), hydroxytyrosol (HyTyr), tyrosol (Tyr), lutein (Lut), verbascoside (Ver), and rutin (Rut), showing the deprotonated molecular ion [M − H]^−^ in the main fragments used in the MRM method; Figure S2: HPLC-MS/MS external calibration curves with equation and correlation coefficient R^2^; Table S1: Statistical results from one-way analysis of variance (ANOVA) of the quantitative data of the selected bio-active compounds monitored by using HPLC-MRM methodology. The aqueous extracts of whole and chopped olive leaves were obtained by using three types of water: ultrapure(U), microfiltered (MF), and osmosis-treated (O) water. Highlighted boxes indicate a significant difference with a *p*-value less than 0.001.
