# Supplementary material for: Eco-Friendly Extraction and Characterisation of Nutraceuticals from Olive Leaves
Source: Molecules. 2019 Sep 25;24(19):3481. doi: 10.3390/molecules24193481 (PMC6804189; doi:10.3390/molecules24193481)
Supplement: Supplementary file 1 [file molecules-24-03481-s001.pdf]

Supporting Information

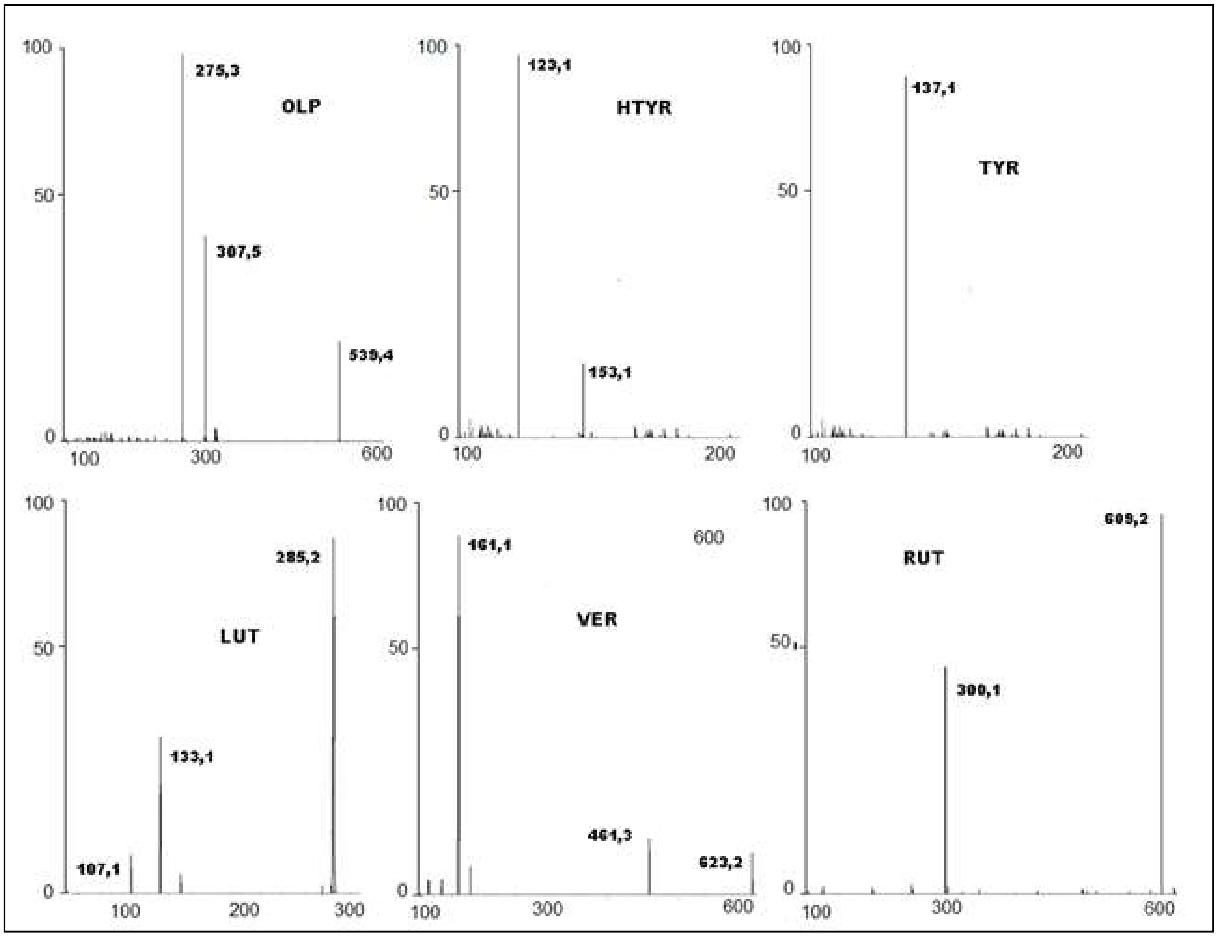

Figure S1. LC-MS/MS spectra of the main phenolic compounds analysed: Oleuropein (Olp), Hydroxytyrosol (HyTyr), Tyrosol (Tyr), Luteolin (Lut), Verbascoside (Ver) and Rutin (Rut) showing the deprotonated molecular ion [M-H]- e the main fragments used in MRM method.

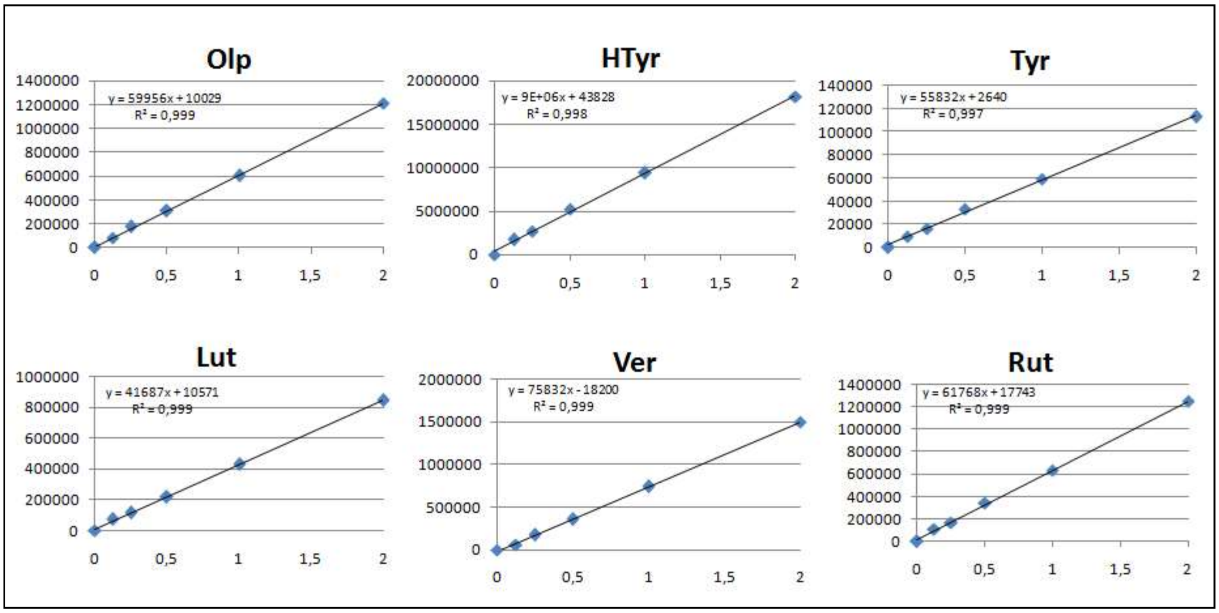

Figure S2. HPLC-MS/MS external calibration curves with equation and correlation coefficient R^2.

Table S1. Statistical results from one-way analysis of variance (ANOVA) of the quantitative data of the selected bioactive compounds monitored by using HPLC-MRM methodology. The aqueous extracts of whole and chopped olive leaves were obtained by using three types of water: ultrapure(U), microfiltered (MF), and osmosis-treated (O) water. Highlighted boxes indicate a significant difference with a *p*-value less than 0.001.

Whole olive leaves

Tukey's pairwise comparisons

Oleuropein

	MF	U	O
**MF**	0	0.0001404	0.9245
**U**	7.349	0	0.0001986
**O**	0.8814	6.468	0

Hydroxytyrosol

	MF	U	O
**MF**	0	0.0008127	0.4747
**U**	5.407	0	0.02548
**O**	1.654	3.753	0

Tyrosol

	MF	U	O
**MF**	0	0.0001068	0.0001068
**U**	19.65	0	0.549
**O**	18.17	1.482	0

Verbascoside

	MF	U	O
**MF**	0	0.001669	0.0001068
**U**	5.075	0	0.0001068
**O**	21.4	26.48	0

Lutein

	MF	U	O
**MF**	0	0.0001068	0.0001068
**U**	26.08	0	0.0001068
**O**	14.64	11.44	0

Rutin

	MF	U	O
**MF**	0	0.0001068	0.0001068
**U**	49.63	0	0.0001068
**O**	13.5	36.14	0

Chopped olive olives

Tukey's pairwise comparisons

Oleuropein

	MF	U	O
**MF**	0	0.0222	0.4285
**U**	3.827	0	0.0005444
**O**	1.765	5.592	0

Hydroxytyrosol

	MF	U	O
**MF**	0	0.0001068	0.8995
**U**	44.13	0	0.0001068
**O**	0.6205	44.75	0

Tyrosol

	MF	U	O
**MF**	0	0.0001084	0.001267
**U**	7.531	0	0.2317
**O**	5.202	2.329	0

Verbascoside

	MF	U	O
**MF**	0	0.0001068	0.0001068
**U**	16.98	0	0.395
**O**	15.13	1.849	0

Lutein

	MF	U	O
**MF**	0	0.0001068	0.0001068
**U**	30.38	0	0.9155
**O**	30.94	0.5665	0

Rutin

	MF	U	O
**MF**	0	0.0001068	0.0001068
**U**	49.09	0	0.9985
**O**	49.17	0.07503	0
